# Biocatalysis: “A Jack of all Trades...”

**DOI:** 10.3390/ijms21145115

**Published:** 2020-07-20

**Authors:** Vladimír Křen, Pavla Bojarová

**Affiliations:** Laboratory of Biotransformation, Institute of Microbiology of the Czech Academy of Sciences, Vídenská 1083, 14220 Prague, Czech Republic; bojarova@biomed.cas.cz

Biotransformation has accompanied mankind since the Neolithic community, when people settled down and began to engage in agriculture. Before that time, “biotransformation” was just a “piece of rotten meat”. Alcohol, accompanying humanity for more than nine millennia, may have been the two-edged sword that helped to drive the development of fermentation technologies, … followed by leavened bread … and then vinegar.

Modern biocatalysis started with “Enzymes” in the second half of 19th century; the term “enzyme” was coined by prof. Kühne [1]. Already in 1858, Pasteur described the first chiral separation of tartaric acid by biotransformation with *Penicillium glaucum* [2]. One of the first patented biotransformation processes—in modern terms—was the production of Neuberg ketol ((*R*)-1-phenyl-l-hydroxy-2-propanone), a precursor of (-)-ephedrin, which is still produced by the same technology [3]. The fulminant development of biocatalytic procedures continued with the industrial production of ascorbic acid (the biotransformation of d-sorbitol into l-sorbose using *Acetobacter suboxydans*); later in the 1950s, biocatalysis started to flourish by steroid-selective bio-oxidation (prednisolon). Then, biotransformations became an indispensable part of our lives, similar to computers and other hi-tech products.

Recent development demonstrates that biotransformation or biocatalysis became a true “Jack of all Trades” as the product portfolio covers virtually all fields of human activities and needs. The Special Issue “Molecular Biocatalysis 2.0” aims to cover a broad array of biocatalytic procedures to demonstrate its versatility and applicability.

A number of papers are devoted to biocatalysis in glycobiology. This is quite logical, since the chemical synthesis of glycostructures still involves demanding multistep cascades of reactions, protection/deprotection steps, and purifications. In contrast, enzymatic systems often provide a single-step selective process, yielding directly a “deprotected” product. This has been nicely demonstrated in the one-pot enzyme cascade synthesis of high molecular weight hyaluronic acid, which is largely used in cosmetic applications [4]. This elegant procedure, an alternative to the use of animal tissues, combines two enzyme modules that in situ generate the respective precursors UDP–GlcA and UDP–GlcNAc using the hyaluronan synthase from *Pasteurella multocida* (*Pm*HAS). This method was optimized to meet the kinetic requirements of *Pm*HAS for high hyaluronic acid productivity and molecular weight. Another glyco-biotechnology applicable in the food industry describes the anchoring of a bacterial chitosanase on the cell walls of *Lactobacillus plantarum*, which is a food-grade expression system [5]. The chitosanase displayed on *L. plantarum* cells is catalytically active and can convert chitosan into chito-oligosaccharides—typically chitobiose and chitotriose as the main products—widely used in pharmacology and medicine. The research groups from Madrid and Prague joined their efforts in a detailed mapping of the acceptor specificity of the transglycosylation reaction catalyzed by β-*N*-acetylhexosaminidase from *Talaromyces flavus*—both as wild type and transglycosidase mutant variants [6]. This study, combined with molecular modelling, deciphered a rather enigmatic problem of acceptor regioselectivity in the transglycosylation reactions and, moreover, provided the first example of the enzymatic glycosylation of an *N*-acetylmuramic acid derivative. The application of biocatalysis in the modification of natural products for the nutraceutics industry was demonstrated in the bioproduction of quercetin and rutinose catalyzed by rutinosidase from *A. niger* [7]. This study also brought a novel concept of “Solid State Biocatalysis”, where both the substrate (rutin) and the product (quercetin) remained in suspension, allowing thus working with concentrations of up to 300 g/L (ca 0.5 M). These results demonstrated for the first time the efficiency of the “Solid-State-Catalysis” concept, which is applicable virtually to any biotransformation involving substrates and products of low water solubility.

Another increasingly attractive research area comprises selective enzymatic redox reactions. Both biooxidations and bioreductions generate new interesting substances, which can hardly be obtained by standard chemical methods. Indigo, a dye used, e.g., for a typical blue color of jeans, is currently produced by a century-old petrochemical-based process. Fraaije et al. [8] showed that the bacterial flavin-monooxygenase from *Methylophaga* sp. can be adapted to improve its ability to convert indole (a commodity chemical) into indigo. This study, entangling computational and structure-inspired enzyme redesign improvement, resulted not only in an upgraded biocatalyst but also provided a better understanding of the structural elements and the detailed mechanism of this important monooxygenase. Oxidases working with inorganic substrates form another interesting facet of biocatalysis. The application of the manganese (Mn^2+^)-oxidizing bacteria *Pseudomonas putida* MnB1 as a whole-cell biocatalyst enabled the effective oxidation of β-keto ester with biogenic MnO_2_, generated in situ, in high yields [9]. On top of that, cells of *P. putida* MnB1 remain alive and are capable of the continuous catalysis of the β-keto ester forming reaction for several cycles. Horseradish peroxidase (HRP) is an important heme-containing oxidase that has been studied for more than a century [10], and it has a vast applicability both in biochemical (ELISA assays) and biotechnological processes. A research group from Vienna produced recombinant HRP in *E. coli* as a fusion protein with quadruple mutations at the glycosylation sites. This construct showed a twice better thermostability and an eight-fold increased catalytic activity with 2,2’-azino-*bis*(3-ethylbenzothiazoline-6-sulphonic acid) as the reducing substrate when compared to the non-mutated recombinant HRP benchmark enzyme [11]. Oxidases can also be used as highly selective sensors. This is the case of glucose oxidase (GO), which is used in electrochemical glucose sensors, e.g., for monitoring and accurate glycemic control for diabetic patient care. Engineered GO was able to catalyze direct single-step modification with a redox mediator (phenazine ethosulfate) on its surface via a lysine residue rationally introduced into the enzyme [12]. This modified GO showed a quasi-direct electron transfer response, which enables its use in the third-generation sensors. It is considered as the ideal solution since the measurements can be performed in the absence of a free redox mediator.

Nitrilases are crucial enzymes for nitrile metabolism in plants and microorganisms. These enzymes have already found broad application in industry—e.g., in the large-scale production of acrylamide from acrylonitrile and in the production of numerous pharmaceuticals [13]. Nitrilases were for the first time described in *Basidiomycota*, and over 200 putative nitrilases were found in this division via GenBank. The representatives of clade 1 and 2 (Nit*Tv*1 from *Trametes versicolor* and Nit*Ag* from *Armillaria gallica*, respectively) and a putative CynH (Nit*Sh* from *Stereum hirsutum*) were overproduced in *E. coli*, and their substrate specificities were analyzed in detail [14]. This study substantially broadens the repertoire of nitrilases available for biocatalytic applications.

The above examples clearly demonstrate that molecular biocatalysis is a pluripotent methodology, which strongly contributes with its inherent green concept to the sustainability of our daily lives. Its nickname “A Jack of all Trades” is definitely not an overstatement.
